# Trends and hotspots in autoimmune hepatitis research: based on bibliometric analysis

**DOI:** 10.1186/s41065-025-00477-6

**Published:** 2025-07-01

**Authors:** Ruisi Li, Yue Xu, XiaoYing Xu, YiHeng Xu, Haitang Huang, Xiaojuan Lv, Chu Liao, Junqiu Ye, Bo Liu, Hengfei Li

**Affiliations:** 1 Hubei University of Chinese Medicine, Hubei Province, Wuhan, 430065 China; 2 Department of Hepatology, Hubei Key Laboratory of the Theory and Application Research of Liver and Kidney in Traditional Chinese Medicine, Hubei Provincial Hospital of Traditional Chinese Medicine, Wuhan, Hubei Province 430061 China; 3 Affiliated Hospital of Hubei University of Chinese Medicine, Wuhan, Hubei Province 430061 China; 4 Hubei Sizhen Laboratory, Wuhan, 430065 China; 5 National Traditional Chinese Medicine Disease Prevention and Control Base, Hubei Province, No. 188 Tanhualin, Wuhan, 430074 China

**Keywords:** Autoimmune Hepatitis, Bibliometrics, Knowledge Map

## Abstract

**Background:**

Autoimmune Hepatitis (AIH) is a chronic inflammatory liver disease characterized by a sustained inflammatory response in the liver, usually associated with abnormalities in the immune system.The purpose of this study was to utilize bibliometric analysis to assess the current state of research on AIH and to predict future research areas and emerging trends.

**Objective:**

In this study, we used bibliometric analysis to comprehensively analyze the literature on Autoimmune Hepatitis (AIH) published during the past two decades.

**Methods:**

Literature from 2003 to 2023 was retrieved from the Web of Science Core Collection, the world's leading citation indexing database. Visualization and analysis were performed using VOSviewer 1.6.18 and CiteSpace 6.2.R3 software.

**Results:**

The study covered 6,390 papers by 28,037 authors from 291 institutions in 110 countries. AIH research output grew significantly, peaking in 2023. The US was the top contributor, followed by China and Japan. Notable institutions included the University of London, Mayo Clinic, and King's College Hospital NHS Foundation Trust. AIH research spans medicine, healthcare, clinical sciences, molecular biology, immunology, etc.

**Conclusion:**

In this paper, we have used bibliometric analysis to systematically review and analyze the research literature on autoimmune hepatitis (AIH) over the past two decades. This comprehensive overview aims to provide scholars dedicated to this field with a clear research lineage and future research directions.

## Introduction

Autoimmune hepatitis is a liver disease characterized by a T-cell-mediated autoimmune response in genetically susceptible individuals [1]. The prevalence of AIH includes all populations from children to the elderly, with a predominance of females [2]. A study conducted in Japan in 2016 showed that the point prevalence of AIH was calculated to be 23.9 (95% CI, 23.3–24.5) based on the general population in Japan (approximately 127 million in 2016). Of the 8505 index patients, 1618 were male and 6887 were female, with a male-to-female ratio of 1:3.3 [3]. Recent epidemiologic studies have shown an increasing trend in the prevalence of AIH worldwide, especially among male patients [4].

Clinical typing of AIH is mainly based on the type of serum autoantibodies and is divided into AIH-1 and AIH-2. AIH-1 is characterized by the positivity of anti-nuclear antibodies (ANA) and/or anti-smooth muscle antibodies (SMA), whereas AIH-2 is characterized by the positivity of anti-hepatorenal and renal microsomal antibodies (LKM-1) [5]. Currently, first-line treatment for AIH consists primarily of corticosteroids (e.g., prednisone) and azathioprine (AZA). These drugs suppress the overreaction of the immune system and reduce liver inflammation. However, long-term use of these drugs may cause certain side effects, and some patients may relapse after stopping the drugs, requiring long-term maintenance therapy or a change in treatment regimen [6]. The pathogenesis of AIH is complex, involving T cells, macrophages, and plasma cells (PCs), and is associated with hepatocellular injury due to inflammatory immune responses [7]. Therefore, although great progress has been made in the epidemiology, diagnosis, pathogenesis, and treatment of AIH, there are still gaps and uncertainties in some areas, and there is no literature that comprehensively reports on the hotspots and development trends of autoimmune hepatitis-related research.

Bibliometrics, a statistical approach to the study of scholarly publishing introduced by Alan Pritchard in 1969, uses statistical data to describe publishing trends and emphasize relationships between published works [8, 9]. It combines mathematics, statistics, and cataloging to quantify and visualize research trends. This approach has become a key method for assessing the quality and impact of scholarly works [10]. In this study, we used two software tools, VOSviewer 1.6.18 and CiteSpace 6.2.R3, to map scientific knowledge and conduct an in-depth study of publications in the field of autoimmune hepatitis (AIH) for the period from 2003 to 2023. Through this approach, we were able to provide a comprehensive overview of the trends in AIH research over the past 20 years, gain a basic understanding of its development process and research hotspots, and predict future research directions, providing valuable ideas and references for scholars in this field. The Tableau Desktop software was used to present the differences in publication volume between the countries.

## Materials and methods

### Data sources and searches

**Fig. 1:**
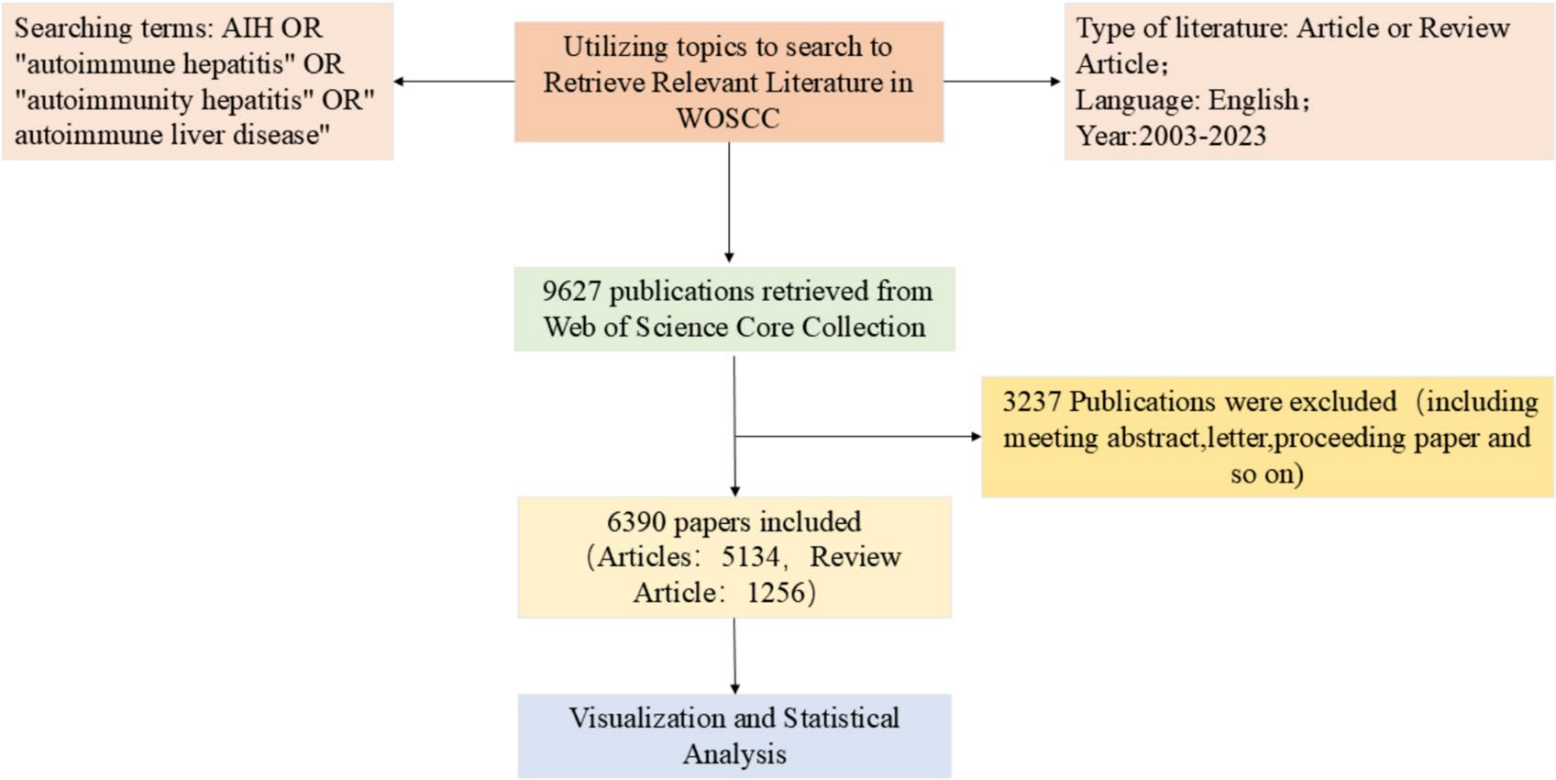
Flowchart for including and excluding publications

The study was searched using the Web of Science Core Collection (WoSCC), one of the world's most influential databases of scientific literature., with a timeframe of January 1, 2003, to December 31, 2023, and a retrieval date of September 1, 2024, with a language restriction of English and an article type restriction of Article or Review Article. Based on Boolean logic operators, in Web of Science, the search formula used was TS = (AIH OR"autoimmune hepatitis"OR"autoimmunity hepatitis"OR"autoimmune liver disease"). To determine the accuracy of the results of the literature dosage analysis, we examined all publications retrieved through the above search strategy by title, abstract, and year of publication. Exclusion criteria were as follows:(i) not related to autoimmune hepatitis, (ii) non-article document types (e.g., reviews, editorial material, letters, and conference abstracts), (iii) duplicate publications, and (iv) non-English publications. In this study, data were retrieved and exported directly from the WoSCC database without the need to apply for ethical approval. We finally identified 6,390 publications, and the detailed screening process is shown in Fig. 1.

### Data analysis

We first exported the retrieved articles in plain text format for the literature analysis to ensure that all complete records and references were included. These files were named"download_XXX.txt", where"XXX"stands for a specific identifier. Next, we imported these text files into two software programs, VOSviewer 1.6.18 and CiteSpace 6.2.R3, for more in-depth analysis.VOSviewer is a tool for constructing and visualizing bibliometric networks, capable of generating various types of network diagrams, such as collaborative networks, co-citation networks, and co-terminology networks. CiteSpace software, developed by Prof. Chaomei Chen, is a Java-based citation visualization software dedicated to bibliometric analysis. By constructing a knowledge graph, CiteSpace can help us identify potential research hotspots and trends within a specific research area. In addition, we use Microsoft Office Excel 2018 to manage the information in the WoSCC database. With Excel, we can effectively analyze and visualize annual publications, allowing us to observe changes in research trends and patterns more clearly.

## Results

### Annual growth trend in publications

**Fig. 2:**
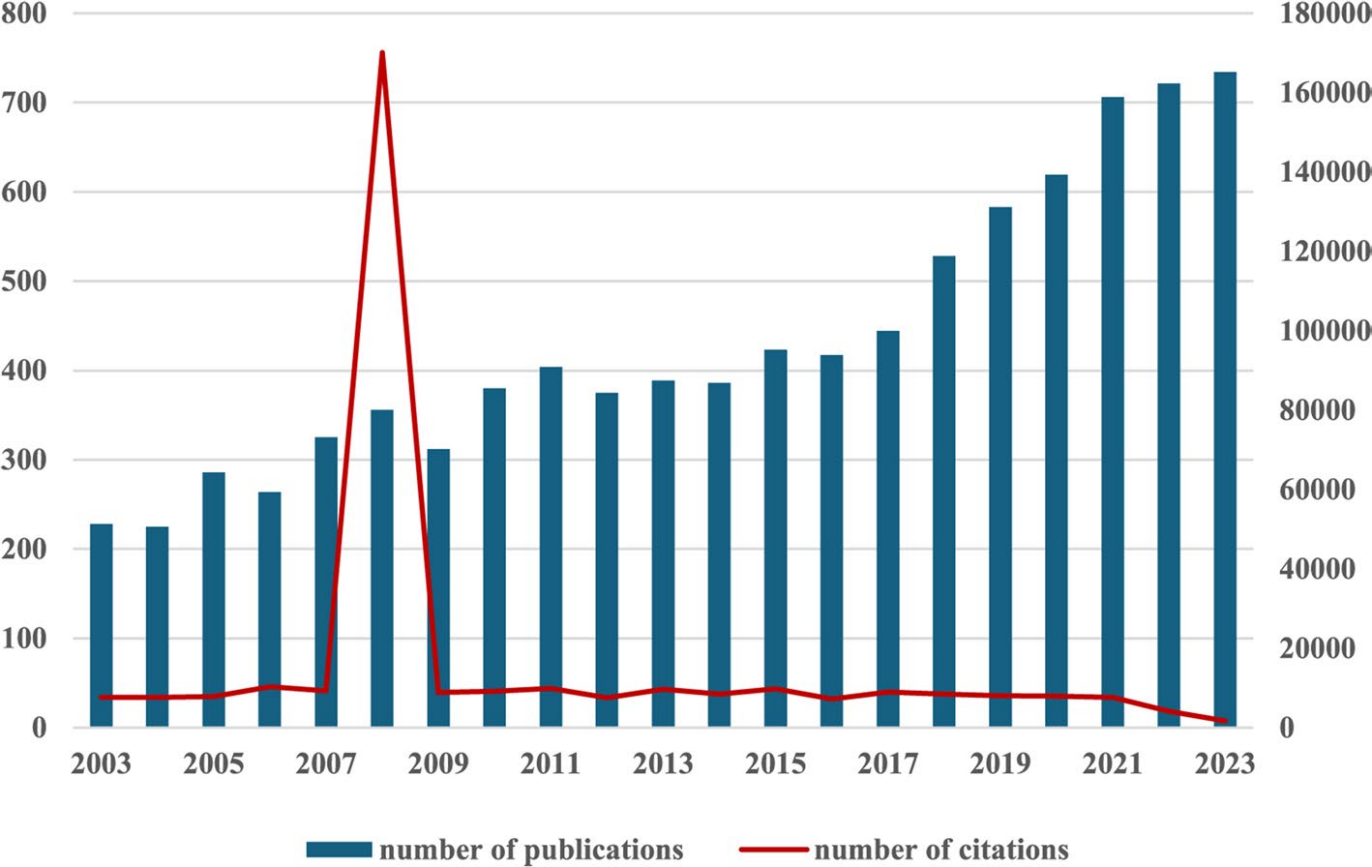
2003—2023 Annual Publications and Citations in Autoimmune Hepatitis

Based on the set data selection criteria, we conducted a literature search in the WoSCC database from 2003 to 2023 and collected a total of 6,390 records related to the research topic. These records contained 5134 Articles (80.3% of the total) and 1256 Review Articles (19.7% of the total). By analyzing these data, we found that the number of publications in the field has shown a trend of continuous growth over time (as shown in Fig. 2), especially in 2023, when the number of articles reached a peak (734), a phenomenon that indicates a rapid growth of interest and research activities in the field of study, and signifies that the field has entered into a new phase of rapid development. At the same time, the number of citations of articles in this field reached a peak in 2008, and the most cited article was “Simplified criteria for the diagnosis of autoimmune hepatitis” published in the journal Hepatology. The most cited article was “Simplified criteria for the diagnosis of autoimmune hepatitis” published in the journal Hepatology, with a total of 1,247 citations, an average of 73.35 citations per year. The article proposes a set of simplified diagnostic criteria that were developed to address the fact that the previous diagnostic criteria proposed by the International Autoimmune Hepatitis Group (IAIHG) were too complex and used primarily for scientific research. The new criteria include factors such as gender, age, autoantibodies, immunoglobulins, exclusion of viral hepatitis, and histologic examination [11]. The proposed diagnostic criteria are important for assisting clinicians in diagnosis and improving the treatment of patients with AIH.

### Output countries/areas

**Fig. 3:**
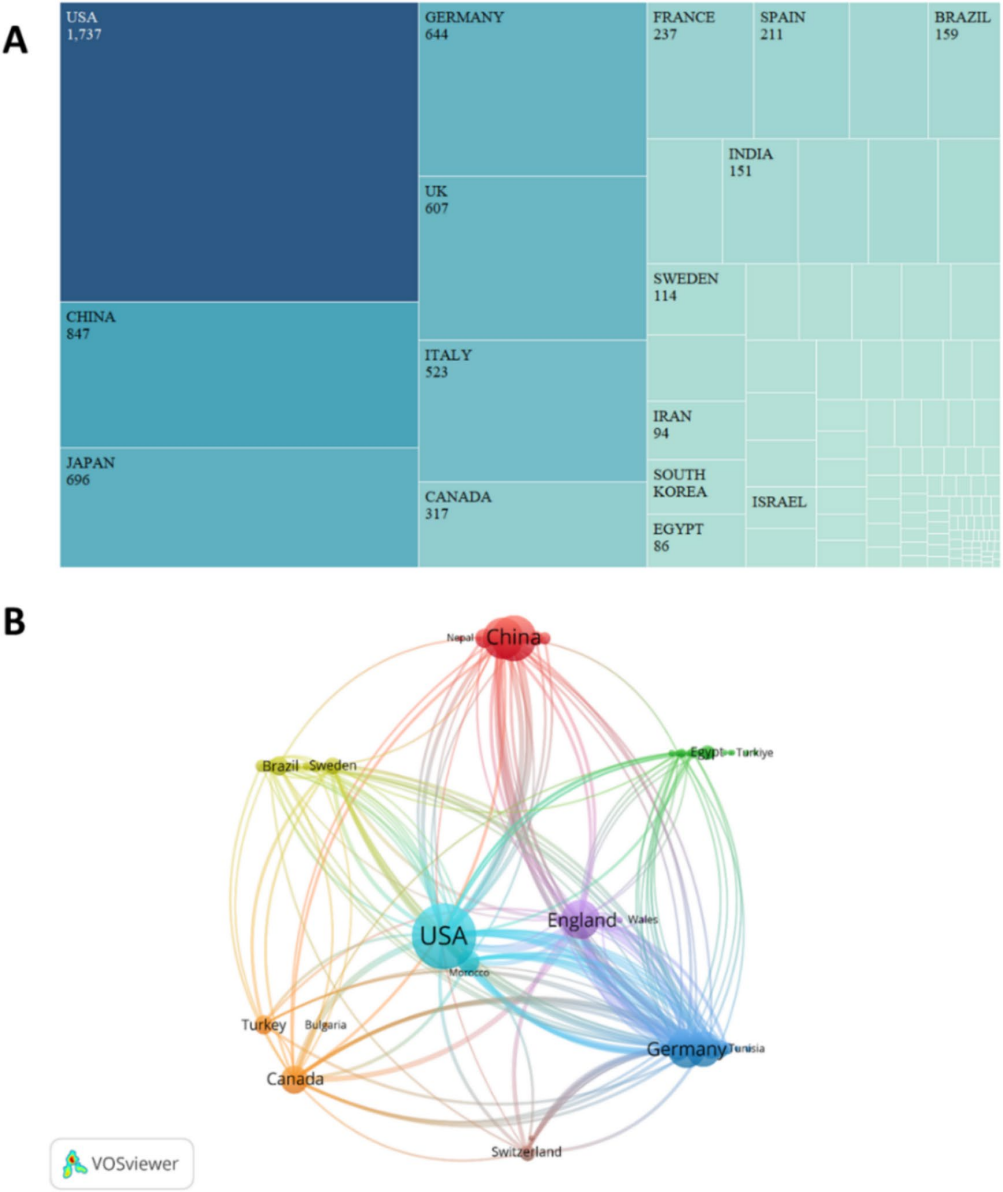
**A** and (**B**) show the number of communications sent by individual countries and how they relate to each other. **A** Distribution of publications by country. **B** Map of country study networks

In analyzing research papers in the field of autoimmune hepatitis (AIH), we note that 291 institutions in 110 countries worldwide are involved in research in this field, Of these, 6,390 met the set inclusion criteria. As shown in Fig. 3A, the United States took the top spot in terms of the number of published papers, with a total of 1,751 studies, or 27.4% of the total, reflecting its leading position in the field of AIH research. Mainland China closely follows it with 863 published studies, or 13.6% of the total, and Japan with 695 published studies or 10.9% of the total. The leading position of the United States may stem from its earliest proposal of immunologic mechanisms and diagnostic criteria for AIH in the 1950s. Ian R. Mackay was one of the first researchers to identify and describe the immunologic mechanisms of AIH, and he first proposed the autoimmune nature of the disease, which provided a solid foundation for AIH research around the world [12]. China has also made notable contributions to the field of AIH. For example, the team of Professors Ruqi Tang and Xiong Ma delved into the alterations of the gut microbiome in AIH patients by analyzing large-scale samples and suggested the potential of the gut microbiome as a biomarker, which provides a new research direction for the diagnosis and treatment of AIH [13]. Japanese researchers, such as Prof. Ohira Hiromasa's team, used the complement system as a research entry point and proposed that circulating factor H levels may serve as a biomarker of AIH disease severity and relapse, which provides a new perspective for understanding the pathophysiological mechanisms of AIH [14]. To further understand the patterns of collaboration in AIH research in different countries and regions, we performed a network map analysis (as shown in Fig. 3B). The size of nodes in the network map represents the closeness of communication between countries or regions. In contrast, the centrality of nodes greater than 0.1 usually indicates denser connectivity and a higher degree of association. As shown in Table 1, England has a centrality of 0.18 and the United States has a centrality of 0.13, which suggests that these two countries occupy a central position in the international collaborative network of AIH research and dominate global research exchange and collaboration. This mode of cooperation may have facilitated the rapid development and knowledge sharing of AIH research and helped global researchers to work together to address this complex immune-mediated liver disease.

**Table 1:** Top 10 countries/areas in terms of number of AIH publications

Country	Count	Centrality
USA	1751	0.13
China	847	0.02
Japan	696	0.05
Germany	644	0.09
England	607	0.18
Italy	523	0.02
Canada	317	0.03
France	237	0.03
Spain	211	0.04
Netherlands	175	0.04

### Output institutions

**Fig. 4:**
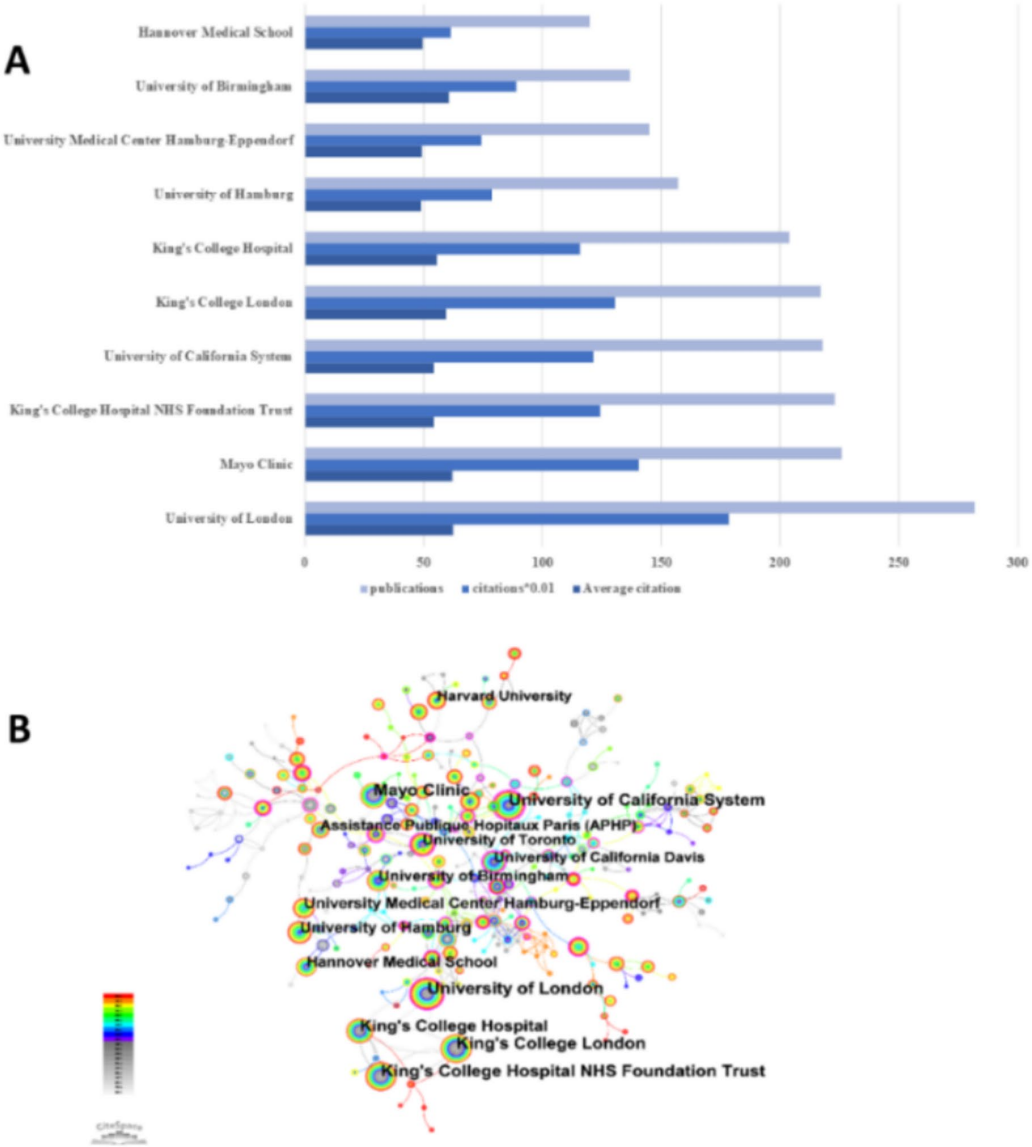
**A** and (**B**) show the issuance status of the various bodies and their interrelationships. **A** Overview of publications, number of citations, and average number of citations by institution. **B**. Network map showing relevant research institutions in the field

A survey of research institutions was conducted using Citespace. A total of 291 institutions were involved in this analysis, with a total of 406 links. The number of publications, citations, and average number of citations for each institution are visualized in Fig. 4A, and Fig. 4B allows for the demonstration of collaborative relationships between different research institutions. By analyzing these collaborative relationships, it is possible to identify which institutions play a central role in the collaborative network, as well as the mode and intensity of cooperation among them. The largest number of publications was from the University of London (*n* = 282, 4.4%), followed in order by the Mayo Clinic (*n* = 226, 3.5%), King's College Hospital NHS Foundation Trust (*n* = 223, 3.5%), and the University of California system (*n* = 218, 3.4%) (Table 2). Based on expert meetings of the International Drug-Induced Liver Injury (DILI) Consortium and the International Autoimmune Hepatitis Group (IAIHG), the University of London, in conjunction with other institutions, summarizes and reports on expert opinion related to drug-induced autoimmune hepatitis (DI-ALH) in 2023, with a focus on the vocabulary of drug-induced autoimmune hepatitis (DI-ALH), diagnostic criteria, treatment, and management, as well as suggestions for future research directions, which not only points out the gaps in current research but also provides important guidance for research and clinical practice in this field [15]. The American Association for the Study of Liver Diseases (AASLD), in conjunction with the Mayo Clinic and others, based on the latest research and evidence, released updated guidelines on the diagnosis and treatment of AIH in 2019, which encompasses a wide range of components such as diagnosis, evaluation, treatment, and prognosis of AIH, and provides a comprehensive guide to the clinical management of AIH [16]. It is noteworthy that five of the top ten research institutions are located in England, demonstrating the high level of interest in AIH research in this country/region. In addition, the centrality of the University of California was as high as 0.44 among the top ten ranked institutions, suggesting that the University of California serves as a bridge for collaboration and communication among institutions.

**Table 2:** Top 10 institutions for autoimmune hepatitis research

Rank	Count	Centrality	Year	Name
1	282	0.12	2003	University of London (England)
2	226	0.02	2003	Mayo Clinic (USA)
3	223	0	2003	King's College Hospital NHS Foundation Trust (England)
4	218	0.44	2003	University of California System (California)
5	217	0	2003	King's College London (England)
6	204	0.1	2003	King's College Hospital (England)
7	157	0.04	2005	University of Hamburg (German)
8	145	0.03	2005	University Medical Center Hamburg-Eppendorf (German)
9	137	0.07	2003	University of Birmingham (England)
10	120	0.01	2003	Hannover Medical School (German)

### Productive and co-cited journals

**Fig. 5:**
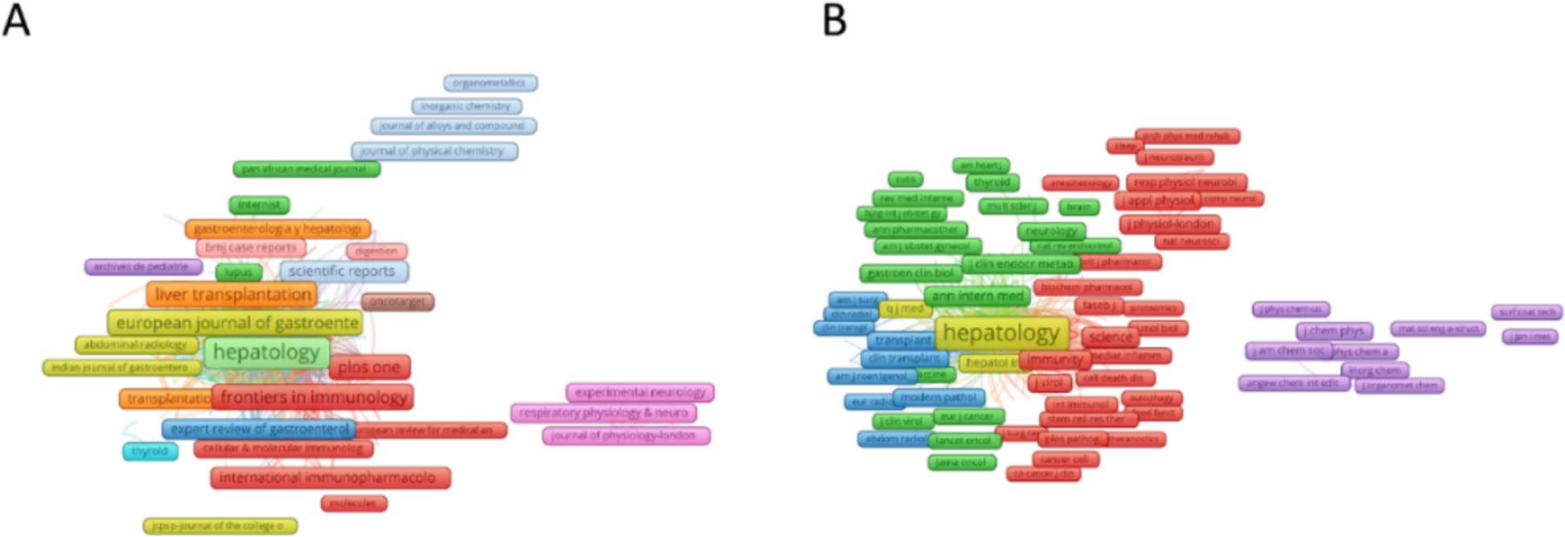
**A** and (**B**) show the number of publications and citations for each journal. (A)Network map showing journals with many papers published in the field. (**B**) Network map showing journals with a high number of citations in this field

To observe the journals with the highest number of publications and co-citations in the treatment of liver fibrosis, co-cited journal analysis was performed using VOSviewer 1.6.18 and CiteSpace 6.2.R3. The statistical results showed that 13,795 journals published literature related to autoimmune hepatitis (Fig. 5A), and Table 3 shows the top ten journals with the highest number of publications related to autoimmune hepatitis. Among these journals, the journal with the highest number of publications was Hepatology (*n* = 155), followed by World Journal of Gastroenterology (*n* = 151), Liver International (*n* = 150), Journal of Hepatology (*n* = 125), etc. The journal with the highest impact factor was the Journal of Hepatology (IF = 26.8). There were 9 journals with a JCR partition of Q1, 1 journal with a partition of Q2, and 5 journals with an impact factor greater than 5 points.

**Table 3:** Top 10 journals related to autoimmune hepatitis research

Rank	Source	Counts	IF (2023)	JCR (2023)
1	Hepatology	155	12.9	Q1
2	World Journal of Gastroenterology	151	4.3	Q1
3	Liver International	150	6	Q1
4	Journal of Hepatology	125	26.8	Q1
5	Hepatology Research	117	3.9	Q1
6	Digestive Diseases and Sciences	109	2.5	Q1
7	Journal of Autoimmunity	93	7.9	Q1
8	Frontiers in Immunology	87	5.7	Q1
9	European Journal of Gastroenterology & Hepatology	82	2.3	Q2
10	Journal of Pediatric Gastroenterology and Nutrition	82	2.4	Q1

The network diagram of cited journals (Fig. 5B) represents the degree of association between journals. The most frequently cited journal in Table 4 was Hepatology (citation frequency *n* = 29,993), followed by Journal of Hepatology (*n* = 18,163), Gastroenterology (*n* = 11,671), American Journal of Gastroenterology (*n* = 6372), Gut (*n* = 5274), and others. The journal with the highest IF was Lancet (IF = 98.4).

**Table 4:** Top 10 co-cited journals related to autoimmune hepatitis research

Rank	Source	Citations	IF (2023)	JCR (2023)
1	Hepatology	29,993	12.9	Q1
2	Journal of Hepatology	18,163	26.8	Q1
3	Gastroenterology	11,671	25.7	Q1
4	The American Journal of Gastroenterology	6372	8	Q1
5	Gut	5274	23	Q1
6	The New England Journal of Medicine	4875	96.2	Q1
7	Journal of Immunology	4680	3.6	Q2
8	Liver Transplant	4593	4.7	Q1
9	Lancet	4361	98.4	Q1
10	World Journal of Gastroenterology	3674	4.3	Q1

### Analysis of research disciplines

**Fig. 6:**
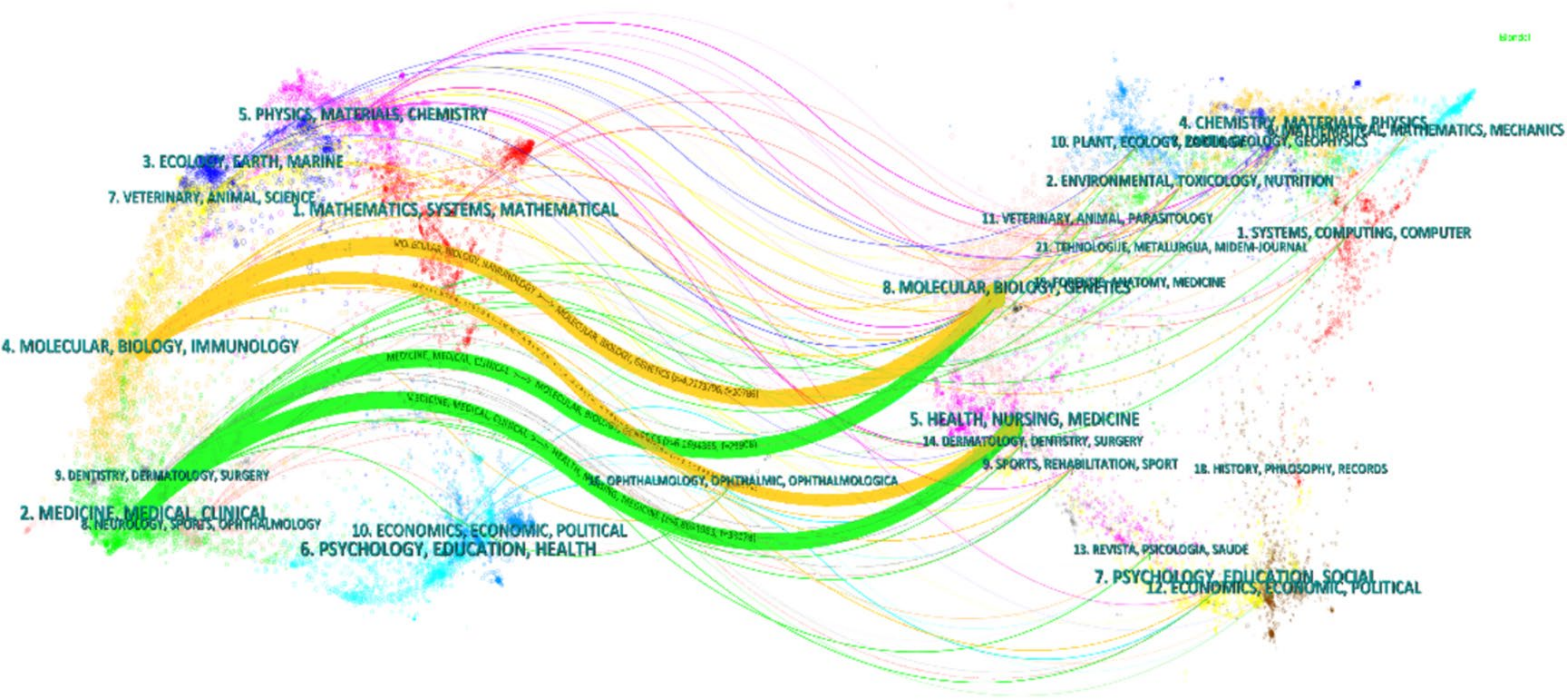
Dual image superimposition of AIH research journals

Figure 6 provides a dual mapping overlay of journal topics. The dual mapping overlay can help identify research trends and hotspots, and by analyzing the changes in the connectivity and clustering of the nodes, future research directions can be predicted. The distribution of journals on the left side represents the main disciplines to which the research area belongs, while the distribution of journals on the right side represents which disciplines are mainly cited in the area, and the Z-score function merges the connecting lines. As described in Table 5, the main disciplines to which the research area belongs are discipline #2 (Medicine, Medical, Clinical) and discipline #4 (Molecular, Biology, Immunology), and the cited journals are mainly distributed in discipline #5 (Health, Nursing, Medicine) and discipline #8 (Molecular, Biology, Genetics). It is also worth noting that publications significantly influence publications in Molecular, Biology, and Immunology (yellow track) in the disciplines of Health, Nursing, Medicine, and Molecular, Biology, and Genetics. In addition, Discipline 5# (Physics, Materials, Chemistry) and Discipline 3# (Ecology, Earth, Oceans) exemplify the diversity and richness of AIH's research areas.

**Table 5:** Correspondence between cited and cited disciplines

Citing region	Cited region	z-score
Medicine, medical, clinical	Health, nursing, medicine	6.869
Medicine, medical, clinical	Molecular, biology, genetics	6.169
Molecular, biology, immunology	Molecular, biology, genetics	4.217
Molecular, biology, immunology	Health, nursing, medicine	2.054

### Highly productive authors and co-cited authors

**Fig. 7:**
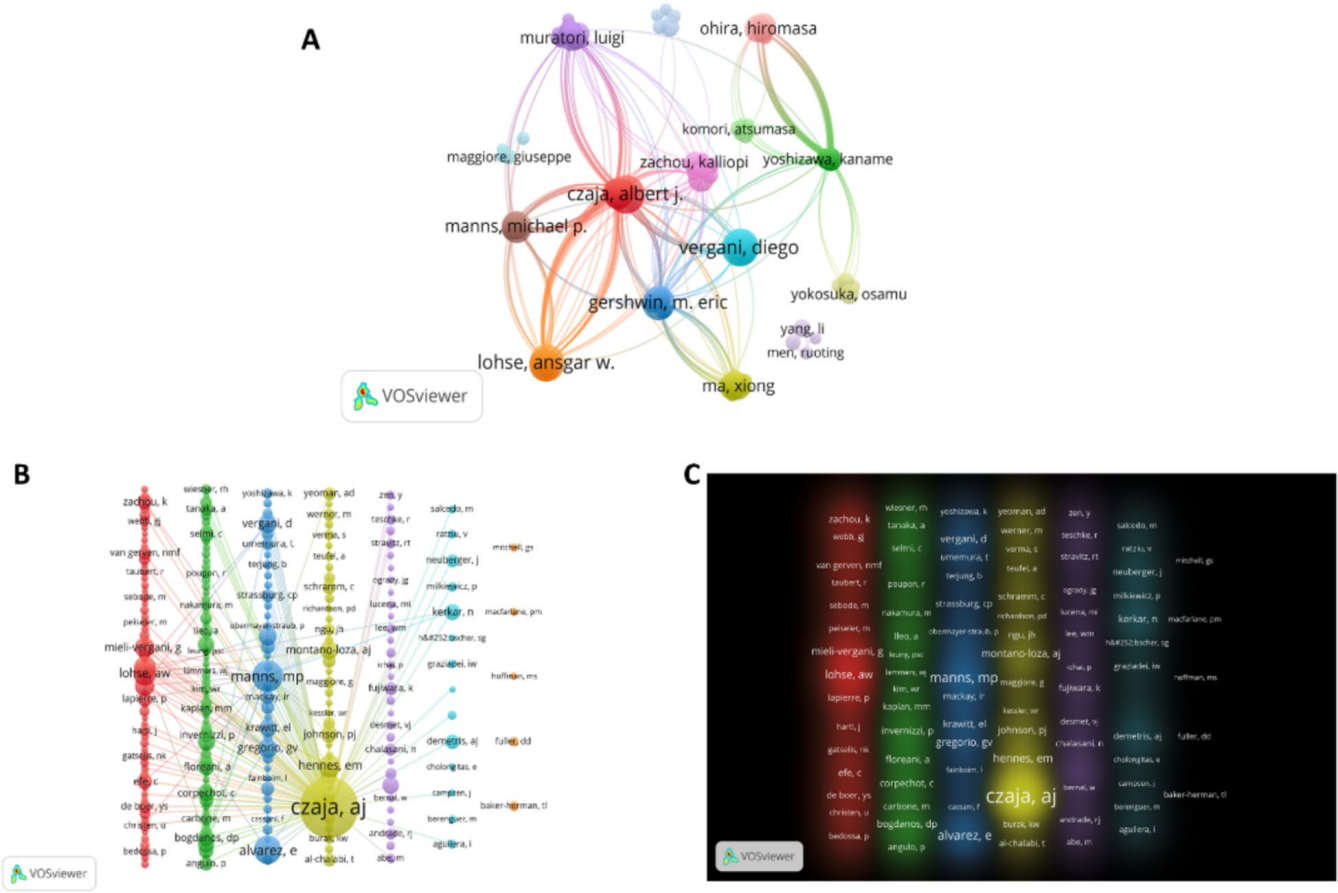
**A**, (**B**) and (**C**) show the authors'publications, citations, and connections to each other. **A** Web map showing authors with a high number of publications in the field. **B** Web map showing authors with more citations for articles in the field. **C** Density plot showing authors with more citations for articles in this field

The results of VOSviewer-based bibliometric analysis showed that 28,037 authors were retrieved in autoimmune hepatitis. Meanwhile, 222 authors were finally selected for network mapping based on a selection threshold of 10 for the minimum number of authors'literature (as shown in Fig. 7A). In addition, Table 6 lists the top 10 authors who showed high productivity and high citation rates in the field of AIH. The author with the highest number of publications is Czaja, AJ who published 93 papers, followed by Vergani, D, Lohse, AW, and Mieli-vegan, G who published 89, 85, and 85 papers respectively. Czaja, AJ is a major scholar in the field of hepatology in the United States. His contributions to autoimmune hepatitis include the systematic study of the pathological mechanisms of AIH, refinement of the diagnostic criteria for AIH, analysis of the roles of different medications in the treatment of AIH, and the first proposal of the concept of “overlap syndrome”, etc. His research has profoundly influenced the global clinical practice of AIH. His research has profoundly influenced the clinical practice of AIH worldwide [17, 18].

**Table 6:** Top 10 authors and co-cited authors associated with autoimmune hepatitis research

Rank	Author	Count	Co-cited author	Citations
1	Czaja, AJ	93	Czaja, AJ	6673
2	Vergani, D	89	Manns, MP	1882
3	Lohse, AW	85	Alvarez, E	1506
4	Mieli-Vergani, G	85	Lohse, AW	1095
5	Gershwin, ME	76	Hennes, EM	1014
6	Schramm, C	68	Longhi, MS	1005
7	Manns, MP	64	Mieli-Vergani, G	818
8	Hirschfield, GM	55	Liberal, R	813
9	Invernizzi, P	50	Gregorio, GV	752
10	Dalekos, GN	49	Montano-Loza,AJ	697

Co-cited authors are two or more authors who are simultaneously co-cited in a series of studies. Co-citation analysis was performed using VOSviewer to illustrate authors with more than 100 citations demonstrating functional and topical impact in the field, and a total of 242 authors were selected (as shown in Fig. 7B). As can be seen through Table 5, Czaja, AJ is both the author with the highest number of publications and the author with the highest number of citations, proving to have a high impact in the field. The high-frequency co-cited authors can be well shown by plotting the density graph, following the gradient of colors (as shown in Fig. 7C).

### Keyword co-occurrence analysis

**Fig. 8:**
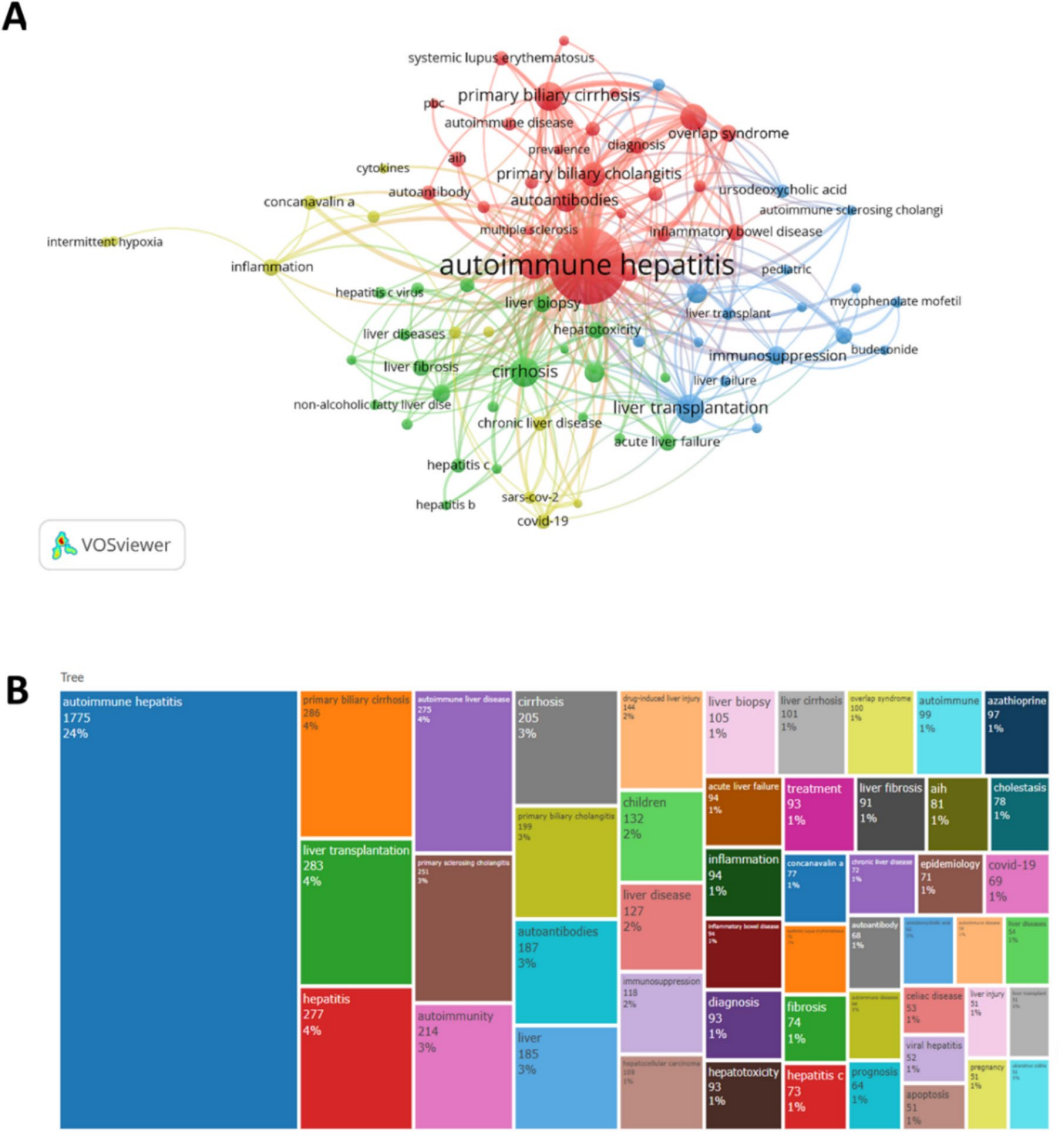
**A** and (**B**) show the categories and distribution of keywords in the field. **A** Web map showing keywords with many publications in the field. **B** Word cloud map displaying keywords

VOSviewer (version 1.6.18) can provide keyword synergy and network clustering analysis. Based on this software, a total of 16,071 keywords were extracted. Considering the small effect on the number of keyword occurrences, we set the “minimum number of keyword occurrences” as our chosen threshold value. In the end, with 30 occurrences as the threshold, 77 keywords met the threshold and were used in the subsequent analysis (as shown in Fig. 8A). These keywords were divided into 4 clusters, and here we mainly analyzed the first 3 clusters, Cluster 1 (red): management of AIH, including epidemiology, treatment, autoantibodies, and differentiation from other autoimmune diseases, such as primary biliary cirrhosis, primary sclerosing cholangitis, etc.; Cluster 2 (green): mechanisms and prognosis of AIH, including cirrhosis, liver fibrosis, chronic active hepatitis, regulatory T cells, etc.; Cluster 3 (blue): treatment of AIH, including liver transplantation, immunosuppression, ursodeoxycholic acid, etc. We visually present the main keywordsw through a word cloud diagram (as shown in Fig. 8B).

### Reference co-citation, clustering, and emergence

Table 7 lists the top 10 most cited papers in the field of AIH, with"Diagnosis and Treatment of Autoimmune Hepatitis"by Michael P Manns et al. having the highest number of citations (*N* = 276). Although published in 2010 by the American Association for the Study of Liver Diseases (AASLD), this article details the diagnostic criteria for AIH, treatment approaches, long-term management strategies, clinical laboratory evaluations, treatment regimens, side effects of treatment, therapeutic endpoints, management of relapse after drug withdrawal, alternative medications, monitoring of hepatocellular carcinoma, indications and outcomes of liver transplantation, and post-transplant AIH. Recurrence and management of new-onset AIH [19].

**Table 7:** Top 10 most co-cited articles in AIH studies

Title	DOI	First Author	Year	Journal	Citations
Diagnosis and management of autoimmune hepatitis	10.1002/hep.23584	Michael P Manns	2010	Hepatology	276
Diagnosis and Management of Autoimmune Hepatitis in Adults and Children: 2019 Practice Guidance and Guidelines From the American Association for the Study of Liver Diseases	10.1002/hep.31065	Cara L Mack	2020	Hepatology	265
EASL Clinical Practice Guidelines: Autoimmune hepatitis	10.1016/j.jhep.2015.06.030	European Association for the Study of the Liver	2015	Journal of Hepatology	246
Simplified criteria for the diagnosis of autoimmune hepatitis	10.1002/hep.22322	Elke M Hennes	2008	Hepatology	227
Autoimmune hepatitis	10.1038/nrdp.2018.18	No authors listed	2018	Nature Reviews Disease Primers	190
Autoimmune hepatitis	10.1056/NEJMra050408	Edward L Krawitt	2006	The New England Journal of Medicine	174
EASL Clinical Practice Guidelines: The diagnosis and management of patients with primary biliary cholangitis	10.1016/j.jhep.2017.03.022	European Association for the Study of the Liver	2017	Journal of Hepatology	137
International Autoimmune Hepatitis Group Report: review of criteria for diagnosis of autoimmune hepatitis	10.1016/S0168-8278(99)80,297–9	F Alvarez	1999	Journal of Hepatology	122
Diagnosis and Management of Pediatric Autoimmune Liver Disease: ESPGHAN Hepatology Committee Position Statement	10.1097/MPG.0000000000001801	Giorgina Mieli-Vergani	2018	Journal of Pediatric Gastroenterology and Nutrition	122
Autoimmune hepatitis–Update 2015	10.1016/j.jhep.2015.03.005	Michael P Manns	2015	Journal of Hepatology	101

**Fig. 9:**
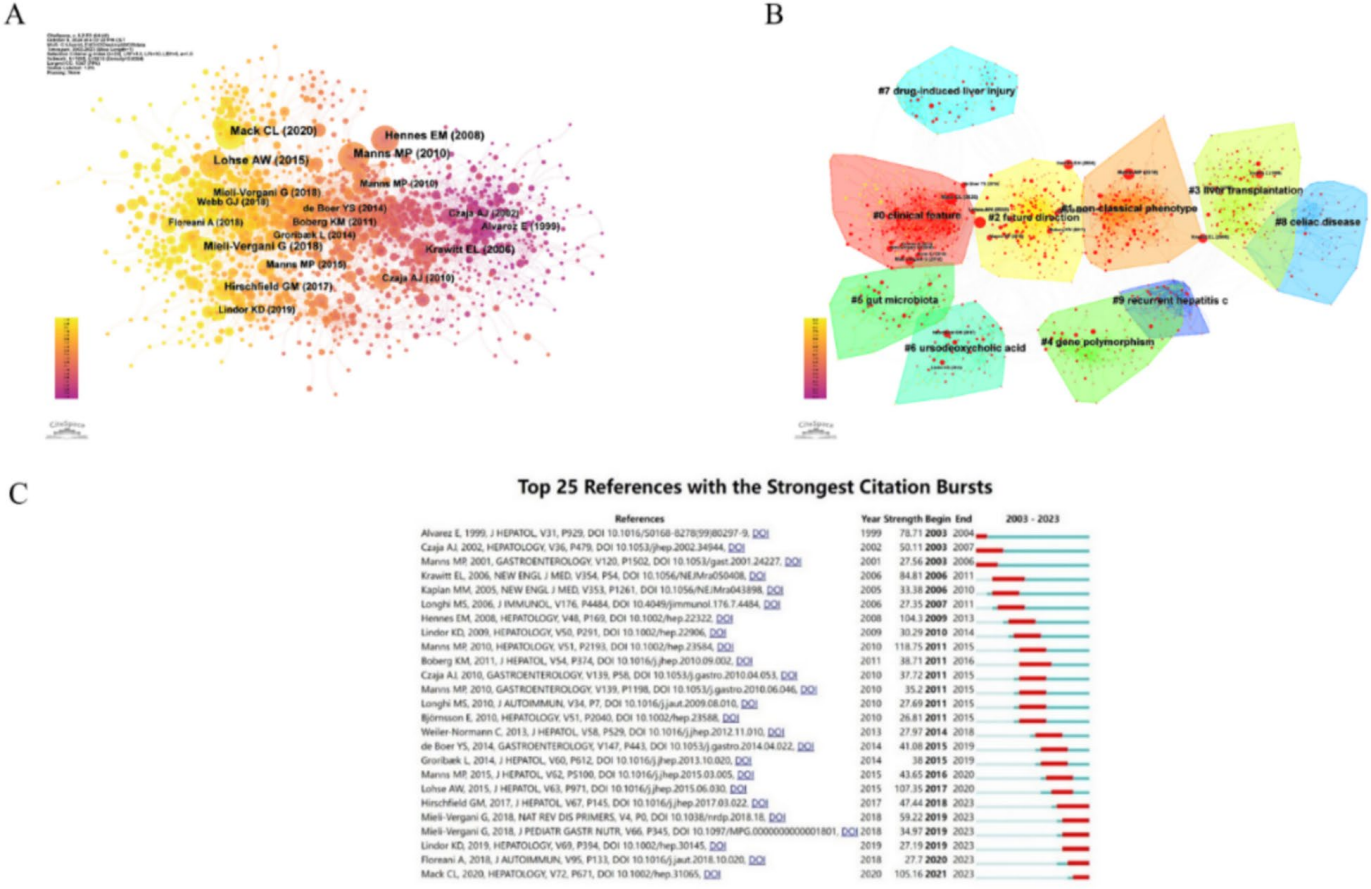
(**A**), (**B**), and (**C**) show the co-citation, clustering, and prominence of references in this field. **A** Visualization of literature co-citation in AIH studies. **B** Clustering of literature co-citations in AIH studies. **C** Top 25 most cited references

The literature was analyzed for co-citation to elucidate the research progress in AIH. The results, as shown in Fig. 9A, show that most of the widely cited literature appeared between 2010 and 2020, especially in 2018, emphasizing the rapid development and remarkable results of this field in that year. Figure 9B shows the clustering of AIH co-cited literature, indicating the thematic categorization of the AIH field. A total of ten clusters are shown in this figure, which are #0 clinical feature, #1non-classical phenotype, #2 future direction, #3liver transplantation, #4gene polymorphism, #5gut microbiota, #6ursodeoxycholic acid, #7drug-induced liver injury, #8celiac disease, etc., demonstrating that these topics have a significant impact on the field. etc., proving that these topics are of high interest in the literature of the field. The analysis of the outbreak of co-cited literature contributes to the study of the duration of the hot content. Figure 9C was plotted to visualize this. The red line in the figure indicates the duration of the outbreak. The highest intensity of outbreaks is the article"Diagnosis and treatment of autoimmune hepatitis"published by Prof. Michael P. Manns in 2010, which was highly cited mainly in 2011–2015, with an outbreak intensity of 118.75; followed by the article"Diagnosis and treatment of autoimmune hepatitis"published by the European Association for the Study of the Liver (EASL) in 2015, which was highly cited mainly in 2011–2015, with an outbreak intensity of 118.75."EASL clinical practice guidelines: autoimmune hepatitis", with an outbreak intensity of 107.35.

## Discussion

Bibliometrics is a science that analyzes literature data through quantitative methods, which can help researchers understand the trends, research hotspots, and academic impact of scientific literature. Visual analytics, on the other hand, transforms this data into graphs and charts, making complex information easier to understand and communicate. Through this method, we can quickly identify trends and key turning points in the research field.

### General information study

The combined results show that the number of publications and citation frequency in this research area of autoimmune hepatitis is on the rise, and is expected to remain an active research topic for some time to come (Fig. 2). The United States, China, and Japan were prominent contributors to this research area, with the United States and England having the highest number of research collaborations with other countries, indicating that these two countries are more deeply involved in this area (Figs. 3A and 3B). The top 10 selected institutions are located in developed countries such as the United States and Germany, which means that developing countries are still lagging in this research area and need to increase their research output (Table 2). By analyzing the frequency and centrality of productive and cited journals, it is possible to identify journals with significant impact in a particular research area, and it can also help researchers to assess the academic impact of different journals, and the journal Hepatology tops the list both in terms of the number of articles published and the number of citations, which proves the unique position of the journal (Fig. 5A, 5B). The results of the analysis of the research disciplines of the published articles show the dynamic evolution of all scientific categories over the last decade, and we found the four most stable and suitable scientific categories in the field of AIH, which have been difficult to study for a long period in the field (Fig. 6). Prof. Czaja, AJ, has made a significant contribution to the diagnostic criteria, treatment, and management of autoimmune hepatitis, with has published a whopping 93 articles with a citation count of 6,673, far exceeding other authors (Figs. 7A and 7B).

### Emerging trends, hot spots and frontiers

By applying keyword cluster analysis, we were able to clearly outline the research framework in the field of autoimmune hepatitis (AIH). This analytical approach allowed us to dig deeper and bring together a wealth of valuable data on the management of AIH, covering the diagnosis, treatment, manifestations, and risk factors associated with the disease. We also conducted a comparative study of the association of AIH with similar diseases such as primary biliary cirrhosis, primary sclerosing cholangitis, chronic active hepatitis, and cirrhosis. The results of these analyses not only pointed us to new trends and hot issues in AIH research, but through co-citation cluster analysis, we further found that the focus of the research was clustered on clinical features, atypical manifestations, post-liver transplantation status, genetic polymorphisms, and gut microbiota of AIH. In the next sections, we will explore in detail the far-reaching impact of these findings on the development of the field, as well as their possible implications for future research directions. This will provide us with a comprehensive perspective to better understand the complexity of AIH and lay the foundation for future scientific exploration.

#### Epidemiology and classification of AIH

AIH is a chronic inflammatory disease of the liver characterized by circulating autoantibodies and elevated serum globulin levels, which lead to the destruction of the liver parenchyma due to loss of tolerance to hepatocytes [20]. According to a population-based national study conducted in the United States from 2014–2019, the estimated prevalence of AIH in the United States is 31.2/100,000 [21]. In Western Europe, the incidence is 0.8—3/100,000, and the prevalence is 11—24/100,000, in Asia, and Japan for example The prevalence rate is between 0.08—0.15 [22]. AIH can be classified into 3 types based on serum autoantibody testing: type 1 is characterized by anti-smooth muscle antibody (SMA) and/or anti-nuclear antibody (ANA) positivity; type 2 is characterized by anti-liver and kidney microsomal antibody (LKM-1) positivity; and the other subtypes can be characterized by anti-soluble liver antigen–antibody/anti-hepatic pancreatic antibody (SLA/LP) Positive [23]. In clinical practice, type I is the most common, and type II AIH accounts for less than 10% of all patients [24].

#### Diagnosis and clinical manifestations of AIH

Autoimmune hepatitis, as an immune-mediated inflammatory liver disease of unknown etiology, does not have a hallmark diagnostic feature; diagnosis requires the presence of a series of typical features; however, due to the presence of individual differences and the complexity of the disease, the same disease can present differently from patient to patient, and it is easily confused with the clinical manifestations of another liver disease [25]. Nearly one-quarter of patients will not present clinical symptoms, and 42% of patients may present with jaundice and itching [26]. The diagnosis of AIH relies on elevated serum transaminase and immunoglobulin G levels, enzymes that can be released into the body's circulation through hepatocellular injury. The presence of autoantibodies and histopathologic manifestations in the liver [27]. Histology is considered a necessary prerequisite for the diagnosis of autoimmune hepatitis, and features of typical AIH include marked portal inflammation, interface hepatitis and varying degrees of lobular hepatitis, plasma cell infiltration, a cobblestone appearance of the hepatocytes, and hepatic rosette knot formation [28].

#### AIH and the overlap syndrome

AIH can co-occur with a variety of liver diseases, here primary biliary cirrhosis is an example, AIH and PBC are both autoimmune liver diseases in which the patient's immune system attacks its liver tissue. The difference is that AIH is mainly an immune response against hepatocytes, whereas PBC as a chronic cholestatic liver disease is characterized by autoimmune destruction of small and medium-sized intrahepatic bile ducts, with a high specificity for antimitochondrial antibodies (AMA) found in 90–95% of patients [29]. Clinically, PBC and AIH can coexist in a syndrome called primary biliary cirrhosis-autoimmune hepatitis (PBC-AIH) overlap. Patients with features of both PBC and AIH develop cirrhosis more rapidly and have a reduced response to ursodeoxycholic acid and steroid therapy, and patients with overlapping PBC and AIH have higher mortality and in situ liver transplantation rates and more cirrhosis-related complications than PBC alone [30]. The exact mechanism of the PBC-AIH overlap syndrome is unclear, and it is difficult to make a definitive diagnosis because patients with the overlap syndrome have different clinical, laboratory, and histologic manifestations. Overlap syndrome should be considered if patients with PBC develop transaminitis and hypergammaglobulinemia, if patients with AIH have cholestasis, or if liver function worsens in patients with well-controlled previous autoimmune liver disease [31].

#### Genetic polymorphisms in AIH

The pathogenesis of AIH is closely related to genetic factors, and specific genetic polymorphisms may influence an individual's susceptibility to autoimmune diseases. A case–control association study of 1,622 Chinese patients with type 1 AIH and 10,466 population-based controls from two independent cohorts found that single nucleotide polymorphisms within the human leukocyte antigen (HLA) region were most strongly associated with AIH [32]. Polymorphisms in the HLA region are the clearest genetic risk factor for AIH. The disproportionate frequency of certain HLA-D alleles in patients with autoimmune hepatitis suggests that these alleles increase the risk of developing the disease, and studies have shown that the positivity rates of HLA-DR3 and HLA-DR4 are higher in patients with AIH than in the general population [33]. In addition to the HLA region, other loci associated with AIH susceptibility also exist, such as cytotoxic T-lymphocyte-associated protein 4 (CTLA-4), a non-HLA susceptibility gene that has been widely studied in autoimmune diseases and is mainly expressed on the surface of regulatory and conventional T cells, inhibiting the response of autoreactive T cells [34]. As a T cell surface molecule that competes with the co-stimulatory molecule CD28, the balance between CTLA-4 and the binding of CD28 to its co-ligand plays an important role in the peripheral control of cellular immune responses [35]. In addition, SH2B3 was identified as the first non-HLA genetic risk factor for AIH through a genome-wide association study of 649 adults with AIH −1 and 13,436 controls in the Netherlands, linking AIH −1 to variants in the MHC region [36].

#### AIH and the gut microbiota

The gut microbiota is a complex ecosystem with a total mass of about 1–2 kg/person. The gut microbiota is responsible for the prevention and elimination of pathogen invasion in addition to maintaining immune system homeostasis and preventing autoimmunity [37]. Gut microorganisms produce a range of metabolites in the host using undigested food and analytes from intestinal epithelial cells as substrates. These metabolites contribute to the onset and progression of hepatic autoimmune disorders by affecting the integrity of the intestinal barrier and are key regulators of host health and disease physiology [38, 39]. The gut and its microbiota interact bi-directionally with the liver through intestinal crosstalk, which exposes the liver to constant exposure to immune cells and microbial products of intestinal origin [40]. A growing body of research suggests that alterations in the gut microbiota are associated with virtually every known liver disease or immune disorder. Patients with AIH have reduced bacterial diversity and altered relative bacterial abundance in the gut microbiota compared to healthy controls [41]. In addition, gut metabolites and immune responses were altered, and changes in the ratio of gut microbiota were involved in AIH by disrupting immune homeostasis as well as activating relevant signaling pathways, such as imbalances in immune cells: imbalances in regulatory T (Treg)/Th17 cells, follicular regulatory T (TFR)/follicular helper T (TFH) cells; short chain fatty acids (SCFAs) and tryptophan; activation of natural killer T (NKT) cells; increased secretion of LPS to stimulate the activation of TLR, NF-κB and other pathways [42, 43].

#### Treatment and prognosis of AIH

AIH as a severe immune-mediated liver disease, leads to several deleterious consequences, including cirrhosis and liver failure [44].AIH has significantly higher short- and long-term mortality rates than the general population, with 65% of early deaths being liver-related [45]. Based on randomized controlled studies, initial treatment for AIH is considered to be steroid-based (usually prednisolone). A second drug, often referred to as a steroid-sparing agent, is often added to this drug in clinical care, with the benefit of achieving similar short-term efficacy to high-dose steroid monotherapy; the most commonly used standard steroid-sparing agent is azathioprine [46]. However, azathioprine is not recommended as first-line therapy because of its potential hepatotoxicity, especially in patients with severe jaundice [47]. Liver transplantation may be considered when patients with AIH present with one of the manifestations of acute liver failure, decompensated cirrhosis, or hepatocellular carcinoma. Autoimmune hepatitis accounts for approximately 5% and 2%−3% of liver transplants in the United States and Europe, respectively [18, 48]. However, liver transplantation is not a one-off solution, and recurrence may still occur after liver transplantation. According to the statistics, the recurrence rate of autoimmune hepatitis is 8%−12% in the first year after liver transplantation, and the recurrence rate is 36%−68% in 5 years [49]. Cyclosporin A alone has now been reported to induce remission for 6 months, followed by maintenance with low-dose prednisone and azathioprine, but studies are still needed to evaluate whether it has comparable therapeutic efficacy to standard treatment [50].

### Limitations and shortcomings

Although this study provides the first comprehensive bibliometric analysis in the field of autoimmune hepatitis, we recognize that there are several limitations in the study that may have influenced its results. First, the data sources for this study were limited to the Web of Science Core Collection (WOSCC) database, which may have limited the breadth of information we obtained. Second, the study was limited to articles and reviews and did not cover other forms of scholarly publications such as conference papers, reports, and monographs. Third, despite our comprehensive search strategy, it is possible that we missed some relevant keywords, which may have some impact on the completeness of the findings. We will continue our efforts to overcome these limitations and provide a more comprehensive perspective in future studies.

## Summary

In summary, the field of AIH research is rapidly evolving and this will continue to be an important area of research. Using bibliometric analysis software, this study analyzes the current state of research in AIH from several aspects. Not only does this study include information on countries, institutions, journals, research disciplines, and authors, but it also specifies the intrinsic relationships between keywords in different clustering groups and their impact on the field. This bibliometric analysis may provide more references for researchers in this field.

## Corresponding author’s profile

Li Hengfei, a male, chief physician, born in July 1979, is the director of the Department of Infectious Diseases of Traditional Chinese Medicine in Hubei Province. He is one of the sixth academic successors of nationally famous old Chinese medicine practitioners. He serves as the vice-chairman of the Youth Committee of the Fifth Infectious Diseases Specialized Committee of the Chinese Society of Integrative Medicine and Western Medicine, and so on. He has presided over and participated in more than 10 provincial and ministerial-level projects, and participated in 2 major national research and development projects. He has published 5 papers in core journals and 5 SCI papers. He was awarded the Hubei Province 2023 Award for Science and Technology Reward Achievement Promotion Award II. She has edited or participated in 3 monographs.

## Data Availability

No datasets were generated or analysed during the current study.
